# A multidimensional comparison of the benefit degrees for patients between ileostomy and colostomy

**DOI:** 10.3389/fmed.2025.1610213

**Published:** 2025-08-04

**Authors:** Yuhang Wang, Lei Cheng, Weichao Li, Zhaowei Gao, Jiayuan Miao, Chunlei Zhang, Zhiqiang Zhu, Feng Zhao, Jing Yi, Chunyuan Liu

**Affiliations:** ^1^The Second Clinical Medical College of Binzhou Medical University (Yantai Affiliated Hospital of Binzhou Medical University), Binzhou Medical University, Yantai, China; ^2^Department of Colorectal and Anal Surgery, Yantai Affiliated Hospital of Binzhou Medical University, Yantai, China; ^3^College of Life Sciences, Yantai University, Yantai, China

**Keywords:** ileostomy, colostomy, enterostomy, complication, patient benefit

## Abstract

**Introduction:**

Enterostomy is widely used in the treatment of various diseases, especially in the treatment of colorectal malignant tumors. It remains to be determined which type of enterostomy can bring more benefits to patients. This study compares the differences in the degrees of benefits for patients between ileostomy and colostomy from multiple dimensions.

**Materials and methods:**

In accordance with the PRISMA statement, the databases such as CNKI, PubMed, Embase, and The Cochrane Library were systematically searched for Chinese and English literatures that were publicly published from 2000 to 2025. The literatures were screened according to the established inclusion and exclusion criteria, and the data were independently extracted by two researchers. The Cochrane bias risk assessment tool was used to evaluate the quality of the literatures. RevMan 5.3 was applied for statistical analysis, including heterogeneity test, calculation of *p* values and odds ratios (OR values), assessment of publication bias, and performance of sensitivity analysis.

**Results:**

A total of 630 studies were initially retrieved, and finally 8 randomized controlled trials were included. The quality of the literatures varied. Most of the studies performed well in key aspects, but some had certain defects. After data combination, the final calculation showed that the odds ratio (OR) was 0.43, with a 95% confidence interval (CI) of [0.28, 0.65], and *p* < 0.0001. The analysis results indicated that within the 95% confidence interval, there were significant differences in the incidence rates of various complications between ileostomy and colostomy. Ileostomy had more advantages in reducing the overall complications of enterostomy.

**Conclusion:**

Ileostomy has more advantages than colostomy in reducing the overall complications of enterostomy surgery. This may be related to factors such as anatomical and physiological characteristics, the nature of intestinal contents, the location of the stoma, and surgical procedures. At the same time, complications can be prevented through multiple measures before, during, and after the operation. However, this study has limitations, such as differences among studies, a short follow-up period, and a relatively small number of included studies and patients.

## Background

Enterostomy can be applied in the treatment of many diseases, such as trauma, inflammation, congenital diseases, benign and malignant tumors, Fournier’s gangrene, and sacral or inguinoscrotal ulcers in chronically frail patients, etc. Among them, enterostomy is most commonly used in the treatment of colorectal malignant tumors. According to epidemiological statistical data, the incidence rate of colorectal cancer ranks second among all cancer incidences (1). Among these patients, a large proportion of them have undergone enterostomy surgery. Various complications may occur after enterostomy surgery. Parastomal hernia often results from poor healing of the abdominal wall muscle layer around the stoma, long-term increased abdominal pressure (such as chronic cough or constipation), or an excessively large abdominal wall opening during surgery, causing intra-abdominal tissues to protrude through the weakened area around the stoma. Mild cases may lead to swelling and discomfort around the stoma, while severe cases can cause intestinal incarceration, resulting in intestinal obstruction or even intestinal necrosis. Stoma ischemic necrosis is mainly caused by impaired blood supply to the intestinal tube during surgery, such as overly tight vascular ligation, excessive tension from intestinal traction, or insufficient blood supply. This leads to darkening and loss of vitality of the stoma mucosa. If not treated promptly, necrotic tissue may cause infection, and in severe cases, reoperation may be required to remove the necrotic intestinal segment and reconstruct the stoma. Stoma stenosis is often caused by excessive proliferation of scar tissue around the stoma or contraction of fibrous tissue during the repair process of injuries such as postoperative infection or ischemia. Stenosis blocks the discharge of intestinal contents, causing abdominal distension and pain, and in severe cases, can lead to complete intestinal obstruction. Stoma prolapse mostly occurs due to loose fixation between the intestinal tube and the abdominal wall, or repeated increases in abdominal pressure (such as severe coughing or heavy lifting), causing the intestinal tube to evert from the stoma. Mild prolapse may only affect appearance and care, while severe cases can lead to edema and ischemia due to compressed blood supply to the intestinal tube, and may even result in irreducible prolapse. Dehydration is more common in patients with ileostomy. Since the ileum is an important site for water absorption, intestinal contents are discharged directly after surgery without reabsorption by the colon, leading to excessive fluid loss. If not replenished in time, symptoms such as thirst, fatigue, and reduced urine output may occur; in severe cases, it can cause electrolyte disorders and kidney damage. Malabsorption syndrome mainly affects patients with ileostomy, especially when the stoma is located near the terminal ileum—a site responsible for absorbing vitamin B12 and bile acids. The rapid discharge of intestinal contents leads to insufficient absorption of nutrients, which may cause weight loss, anemia, osteoporosis, and other issues. In the long term, this can affect the patient’s physical strength and immunity. The intestine is one of the important components of the human digestive system and plays a vital role in human life activities. Therefore, after enterostomy surgery, it is particularly important to rapidly restore intestinal function and prevent complications. However, which type of enterostomy procedure can bring more benefits to patients still remains to be discussed. Moreover, with the increase in the number of colon cancer patients, the number of patients with permanent and short-term stomas has also surged sharply. We must pay more attention to this group and understand the impact of enterostomy on their disease prognosis, economic situation, psychological state, quality of life, and other aspects. This article will elaborate from multiple angles, including prognosis, prevention of complications, patients’ economic burden, surgical safety, patients’ quality of life, etc., and analyze which surgical procedure can bring more benefits to patients.

## Materials and methods

A systematic literature review was conducted following the Preferred Reporting Items for Systematic Reviews and Meta-Analyses (PRISMA) statement. We comprehensively and systematically searched multiple authoritative databases using a computer, including CNKI, PubMed, Embase, The Cochrane Library, etc., to obtain publicly published literatures comparing the incidence rates of complications between ileostomy and colostomy. The search time range was set from the beginning of 2000 to the end of 2025 to ensure the comprehensiveness and timeliness of the literatures. The search terms were formulated by comprehensively considering the types of stomas (ileostomy, colostomy), vocabulary related to complications (such as stoma prolapse, stoma bleeding, anastomotic leakage, parastomal hernia, intestinal obstruction, incision infection, etc.) and vocabulary related to the research types. Boolean logical operators (such as AND, OR) were used for combined retrieval to ensure the accuracy and sensitivity of the retrieval strategy. Then, the references cited in the included literatures were screened. The time limit for the included literatures was from the establishment of the database to the present. In order to prevent missed and false retrievals, a combination of subject headings and free terms was adopted during the retrieval period. The languages of the articles were limited to Chinese and English.

Literature inclusion criteria: ① Literatures involving patients with colon cancer who underwent stoma creation; ② Literatures involving patients with rectal cancer who underwent stoma creation; ③ Literatures from which clear data can be obtained; ④ Literatures with the research indicators being the prognosis of enterostomy and the prevention of complications; ⑤ Randomized controlled trials.

Literature exclusion criteria: ① Literatures not involving enterostomy and its prognosis; ② Literatures from which accurate data cannot be obtained; ③ Literatures in which, although enterostomy is involved during the research process, there are other interfering factors; ④ Literatures not in Chinese or English; ⑤ Duplicate publications and literatures for which the full text cannot be obtained; ⑥ Conference papers.

The retrieved articles were independently screened and the data were extracted by two researchers according to the literature inclusion and exclusion criteria, and cross-checked. In case of any divergence, it was judged through discussion between the two parties or by inviting a third party. The data extraction content included the author, publication year, research subjects, research type, references, and the country of publication. All the data we collected were sourced from the published articles. We used Microsoft Excel to extract the information from each eligible article. This included the article title, author information, publication time, operation time, hospitalization time, the number of patients involved in the study, the types of complications and the number of patients, and all the data were integrated. Among the above-mentioned information, the number of patients with complications, the number of patients who received enterostomy, and the ratio of the two (that is, the incidence rate of enterostomy complications) are the key information required by this study. During the process of reviewing the articles and extracting the data, the two reviewers independently extracted the data and consulted with each other in case of any divergence. If there were situations that could not be resolved through negotiation between the two, the suggestions of a third party would be combined to finally make a decision.

## Literature quality assessment

During the process of literature quality assessment, different quality assessment tools should be used to evaluate the quality of the included literatures according to the different research types of the articles. Since all the studies included in this article are randomized controlled trials, we used the Cochrane Bias Risk Assessment Tool to evaluate the literatures from aspects such as the generation of random sequences, allocation concealment, blinding of participants and researchers, the integrity of outcome data, selective reporting of research results, and other sources of bias. The literature quality is classified into three levels: low risk, high risk, and unclear.

## Statistical analysis

The relevant statistical data of this article were analyzed using Revman5.3. The incidence rates of the two surgical procedures, ileostomy and colostomy, were compared in a binary variable manner.

Heterogeneity Test: The Cochrane Q test and I^2^ statistic were used to conduct a heterogeneity test on the included studies. If *p* > 0.1 and I^2^ < 50%, it indicates that there is little heterogeneity among the studies, and a fixed-effect model is adopted for the combined analysis; if *p* ≤ 0.1 and I^2^ ≥ 50%, it indicates the presence of significant heterogeneity, and the source of heterogeneity is further analyzed, such as through subgroup analysis (grouping according to factors such as the type of patient’s disease and the surgical method), sensitivity analysis and other methods to explore the causes of heterogeneity. If the heterogeneity cannot be eliminated by the above methods, a random-effect model is considered for the combined analysis.

In this article, we will use the M-H test to calculate the *p* value and the odds ratio (OR) value. If, under the condition of *α* < 0.05, the calculated *p* ≤ 0.05, it can be concluded that the probabilities of complications caused by the two surgical methods are different and there is a significant difference. When the 95% confidence interval of the OR does not include 1, it indicates that there is an association between ileostomy and colostomy, and the association is statistically significant. If the OR is greater than 1, it indicates that ileostomy is a risk factor for the occurrence of complications compared with colostomy; if it is less than 1, it indicates that ileostomy is a protective factor for the occurrence of complications compared with colostomy.

Publication Bias Assessment: A funnel plot was used to intuitively assess whether there was publication bias in the included studies. If the funnel plot shows an asymmetric distribution, the Egger’s test or Begg’s test is further used for quantitative analysis to determine whether the publication bias is statistically significant. If there is publication bias, corresponding treatment methods are adopted to correct the results to improve the reliability of the results.

Sensitivity Analysis: Sensitivity analysis was carried out using R 4.4.1 according to the pre-set factors. By excluding a single study one by one, observing the changes in the combined effect size, evaluating the influence of a single study on the overall results, and judging the stability and reliability of the results.

## Results

### Results of article retrieval

**Figure 1:**
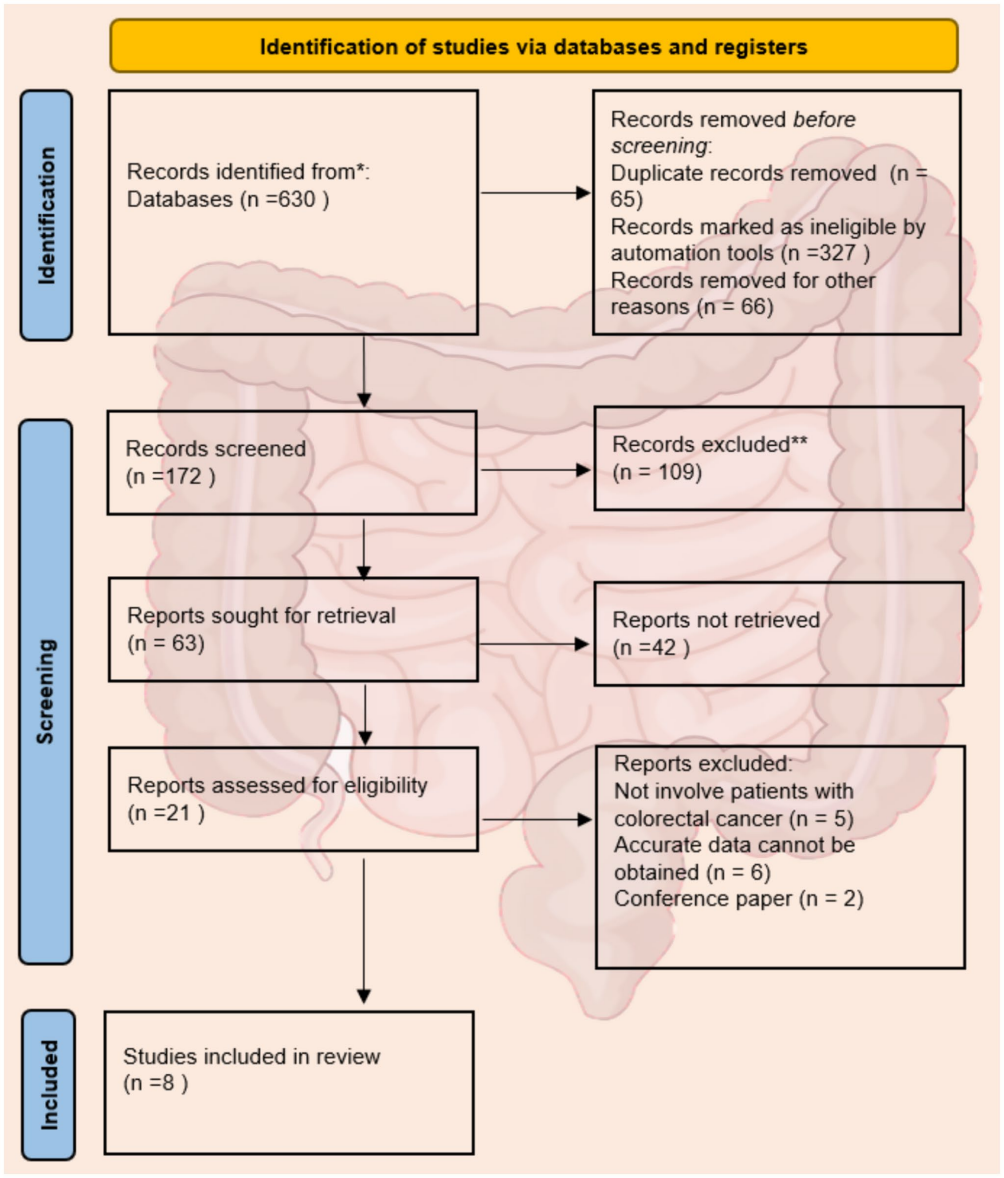
PRISMA literature search flow chart.

After the initial search, we identified 630 studies. After screening with Endnote, there were 565 studies remaining. According to the inclusion and exclusion criteria, through reading the titles, abstracts, keywords, references and some full texts, duplicate studies or those that did not meet the eligibility criteria were excluded (n = 502). The full texts of 63 studies were evaluated, among which 42 studies were excluded because they did not meet the inclusion criteria. Finally, we selected 8 studies and extracted the data on complications after colostomy from all the included studies (Figure 1) (2–9). Table 1 lists the detailed information of the included studies.

**Table 1:** Detailed information of the included studies.

Research	Surgical method	The number of patients	Age ( $\bar{x}$ ± s)	Time of operation (h, $\bar{x}$ ± s)	Postoperative hospital stay (day, $\bar{x}$ ± s)
Wan et al. (2)	LI	46	**70.62 ± 3.57**	**2.60 ± 0.49**	**8.86 ± 1.58**
	LC	42	**70.56 ± 3.61**	**2.54 ± 0.46**	**8.79 ± 1.60**
Wu et al. (3)	LI	49	**60.53 ± 10.67**	**1.32(1.04,1.67)** **M(P25,P75)**	**4.00(3.50,4.50)** **M(P25, P75)**
	LC	50	**61.34 ± 11.12**	**1.54(1.25,2.00)** **M(P25,P75)**	**5.50(5.00,6.00)** **M(P25,P75)**
Gao et al. (4)	LI	40	**68.12 ± 1.33**	**2.74 ± 0.52**	**2.50 ± 0.58(Postoperative fasting time)**
	LC	40	**68.12 ± 1.33**	**2.52 ± 0.61**	**4.61 ± 0.72(Postoperative fasting time)**
Ren et al. (5)	LI	35	**59.86 ± 9.13**	**2.43 ± 0.51**	**8.04 ± 1.63**
	LC	35	**60.22 ± 8.37**	**2.61 ± 0.74**	**8.11 ± 1.54**
Xu et al. (6)	LI	45	**69. 1 ± 5. 8**	**1.24 ± 0.39**	**6.36 ± 1.31**
	LC	45	**69. 7 ± 5. 6**	**2.46 ± 0.43**	**8.87 ± 1.85**
Jin et al. (7)	LI	40	**69.6 ± 5.7**	**2.83 ± 0.87**	**7.4 ± 1.6**
	LC	40	**69.0 ± 6.2**	**2.775 ± 0.63**	**8.5 ± 1.5**
Rullier et al. (8)	LI	107	**Detailed information cannot be obtained.**		
	LC	60			
Law et al. (9)	LI	42			
	LC	38			

The unit of “age” is year(s). “Time of operation” refers to the duration from the start to the end of the surgery, with the unit being hour(s). “Postoperative hospital stay” is the total time spent by patients from the end of the surgery to their discharge. Some studies use patients’ postoperative fasting time to evaluate the speed of their recovery.

### Quality assessment results

A total of 8 randomized controlled trial (RCT) articles related to enterostomy complications were included in this study, and a comprehensive evaluation of their quality was carried out. Most of the articles were relatively rigorous in the research design. They clearly elaborated the research purpose and hypothesis, clearly defined the intervention measures of the intervention group and the control group, and reasonably set the primary and secondary outcome indicators. However, there were still a few articles with a slightly brief description in the research design section. They did not fully explain the specific implementation details of the intervention measures, the course of treatment arrangements, etc., which may affect the reproducibility and generalizability of the research results.

In terms of randomized grouping, more than half of the articles described the randomization method in detail, such as using computer-generated random numbers, random permutation tables, etc., to ensure the randomness and concealment of the allocation of research subjects. However, some articles only briefly mentioned “randomized grouping” without further elaborating on the specific randomization process, making it difficult to judge the quality and reliability of the randomization. This may lead to selection bias and have a potential impact on the accuracy of the research results.

The application of blinding is crucial for reducing bias in research. However, due to the reason that the informed consent rights of patients were involved in the studies included in this article, blinding was not adopted, which may cause the research results to be biased due to the subjective factors of the researchers or research subjects.

Most of the articles reasonably calculated the sample size and provided detailed calculation processes and bases according to factors such as the research purpose, expected effect size, and test power. This provides a strong guarantee for the statistical significance of the research results. However, there were still some articles that did not mention the method of determining the sample size, or the sample size calculation was unreasonable. A too small sample size may lead to insufficient test power and an inability to accurately detect the true effect of the intervention measures.

Most of the articles performed well in the data collection and analysis stage, and they made detailed records of the loss to follow-up and dropout situations. However, there were still a few articles with defects in data management. They did not analyze the reasons for the loss to follow-up deeply enough and did not handle the missing data reasonably, which may cause deviations in the research results.

**Figure 2:**
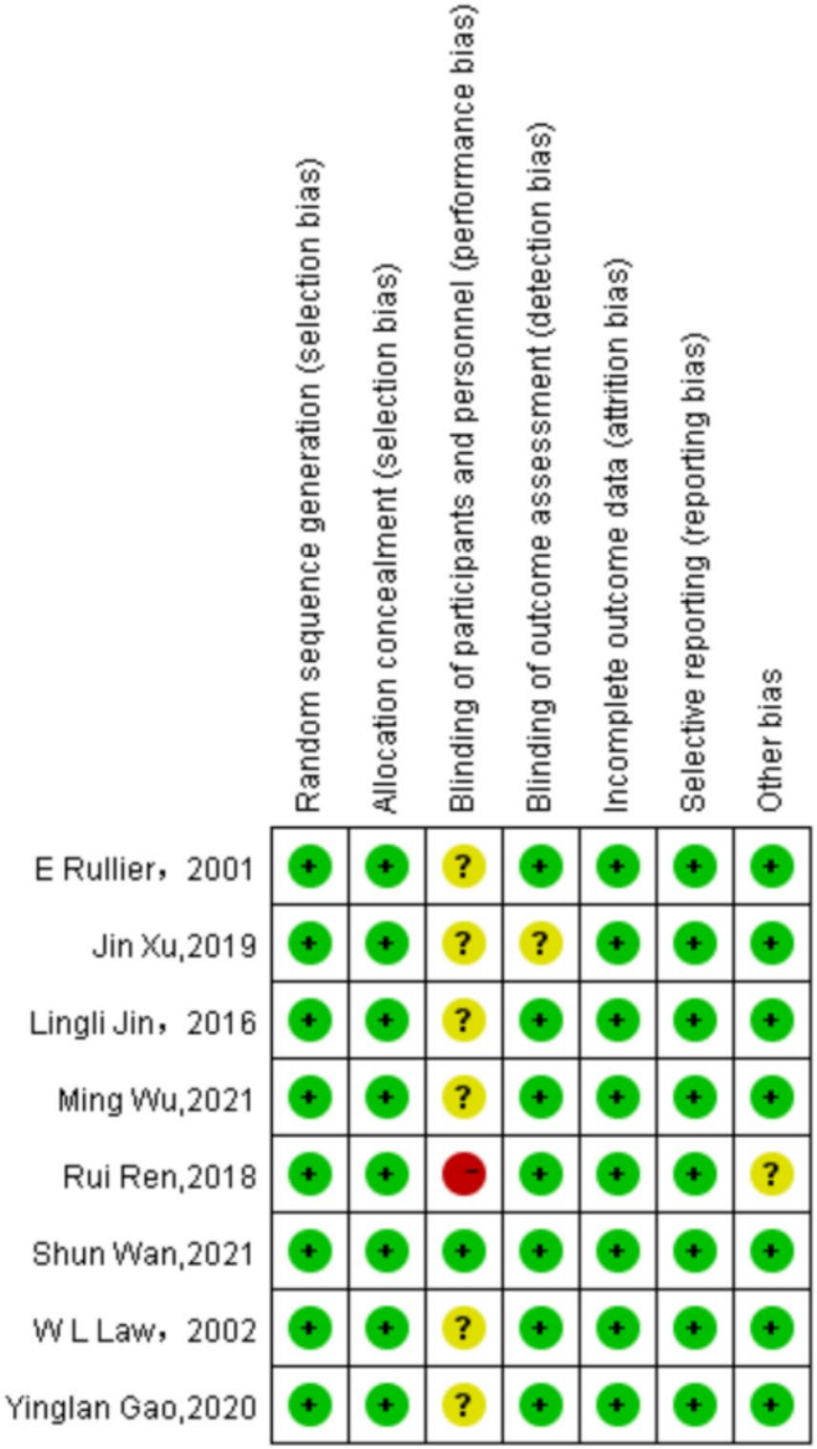
Risk of bias summary: review authors’ judgements about each risk of bias item for each included study.

**Figure 3:**
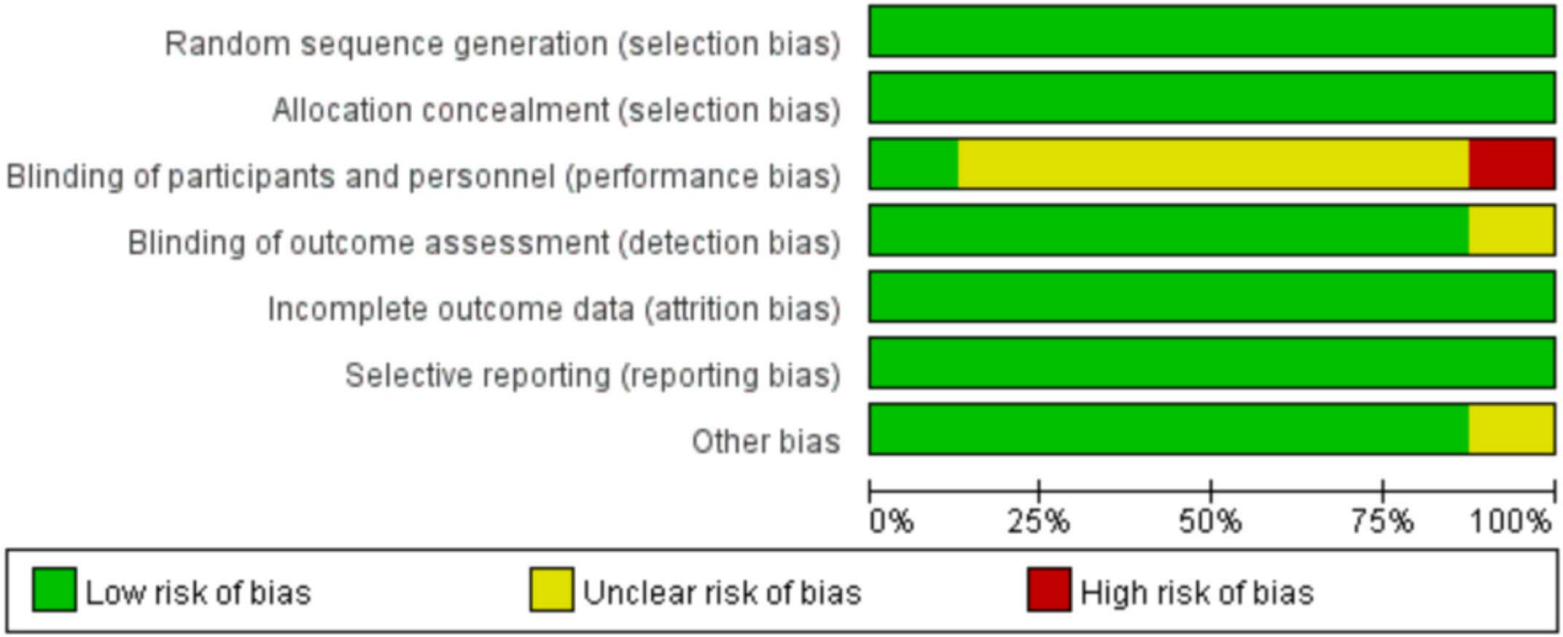
Risk of bias graph: review authors’ judgements about each risk of bias item presented as percentages across all included studies.

Overall, the quality of the included RCT articles related to enterostomy complications varied. Although some articles still had problems such as incomplete descriptions and non-standard methods, most of the articles performed well in key aspects such as research design, randomization, and blinding. Future studies need to make improvements in these aspects to enhance the research quality and the credibility of the results. The quality assessment results are shown in Figures 2, 3.

### Synthesized findings

**Figure 4:**
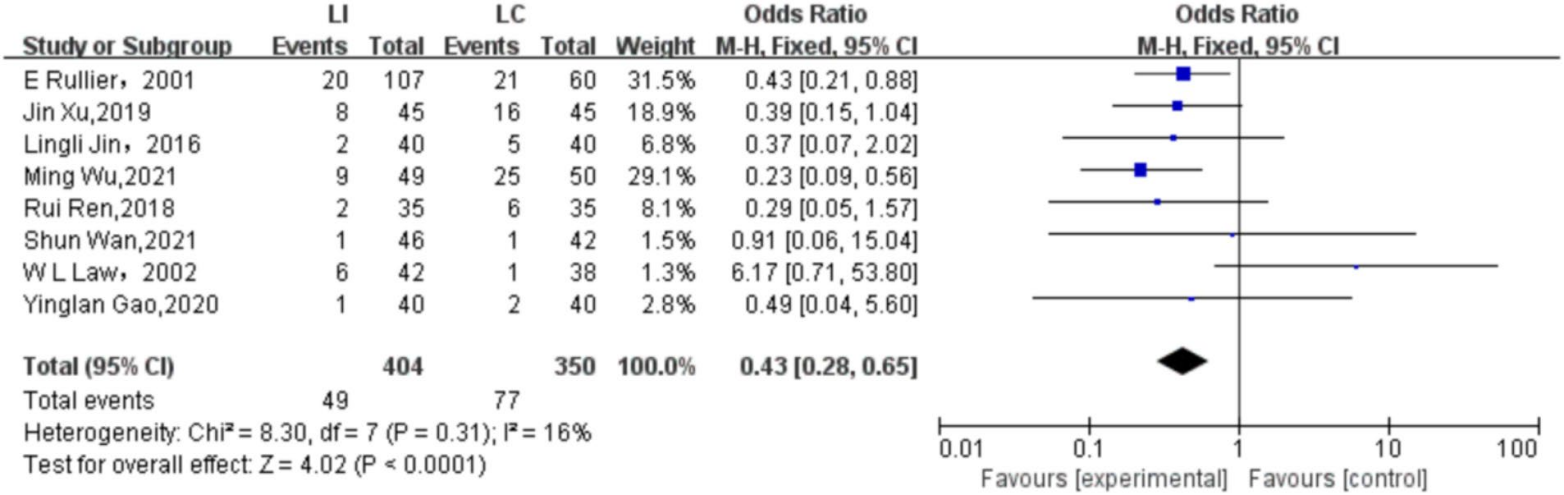
Forest plot of comparison.

**Figure 5:**
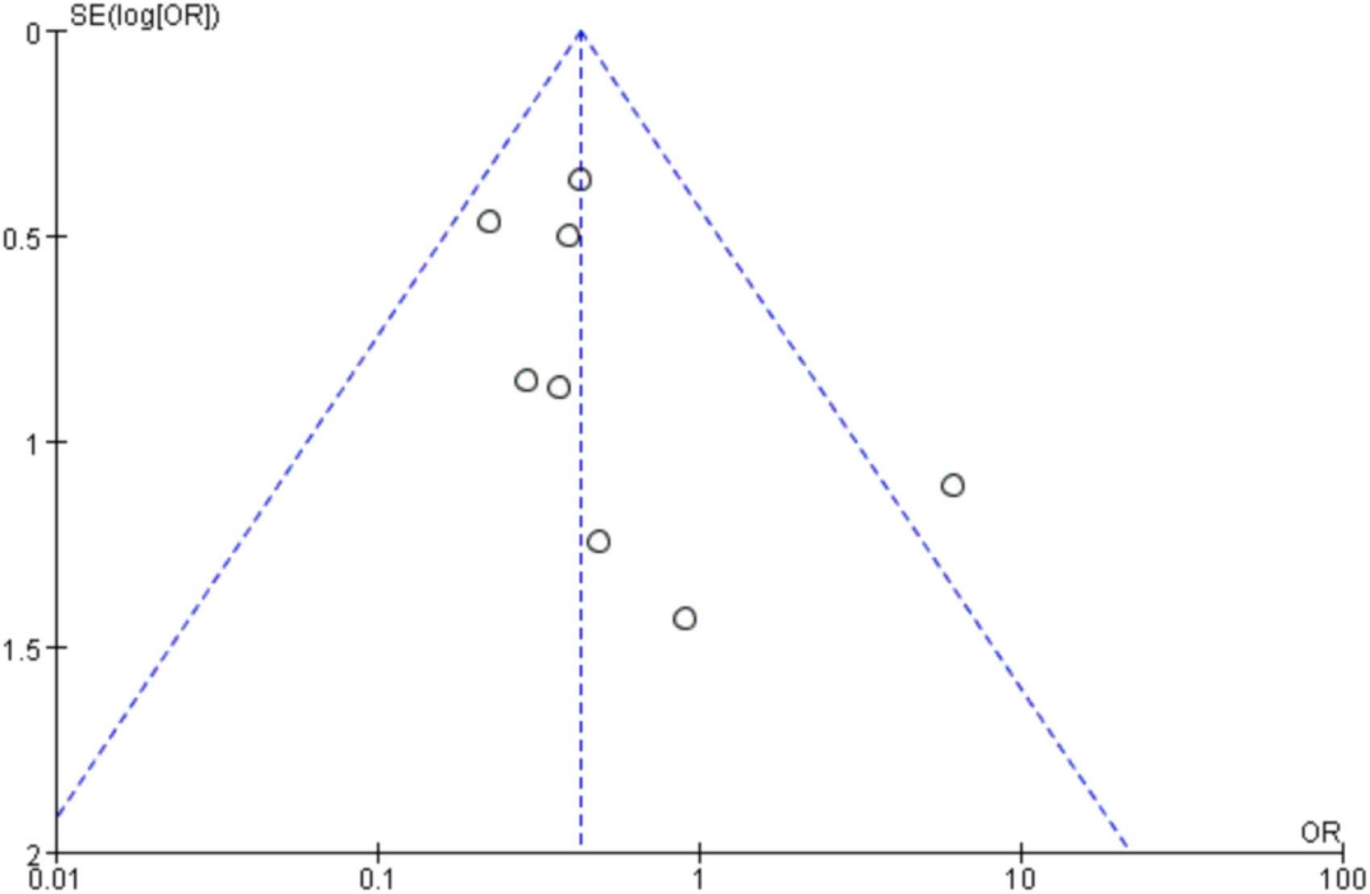
Funnel plot of comparison.

As shown in Figure 4, the figure displays the effect sizes of each study and their confidence intervals, as well as the vertical line and rhombus mark representing the combined effect size, intuitively presenting the distribution of the effect sizes of each study and the combined results. We adopted the Fixed-effects model to conduct a combined analysis of the five included studies, and the method used was the Mantel–Haenszel (M-H) test. Finally, we calculated that the odds ratio (OR) was 0.43, with a 95% confidence interval (CI) of [0.28, 0.65], and *p* < 0.0001. Eventually, we concluded that within the 95% confidence interval, there were significant differences in the incidence rates of various complications between ileostomy and colostomy. In terms of reducing the overall complications of enterostomy, ileostomy (LI) is a favorable factor compared to colostomy (LC). As shown in Figure 5, there was no significant publication bias among the 8 studies included in this article.

### Sensitivity analysis

**Figure 6:**
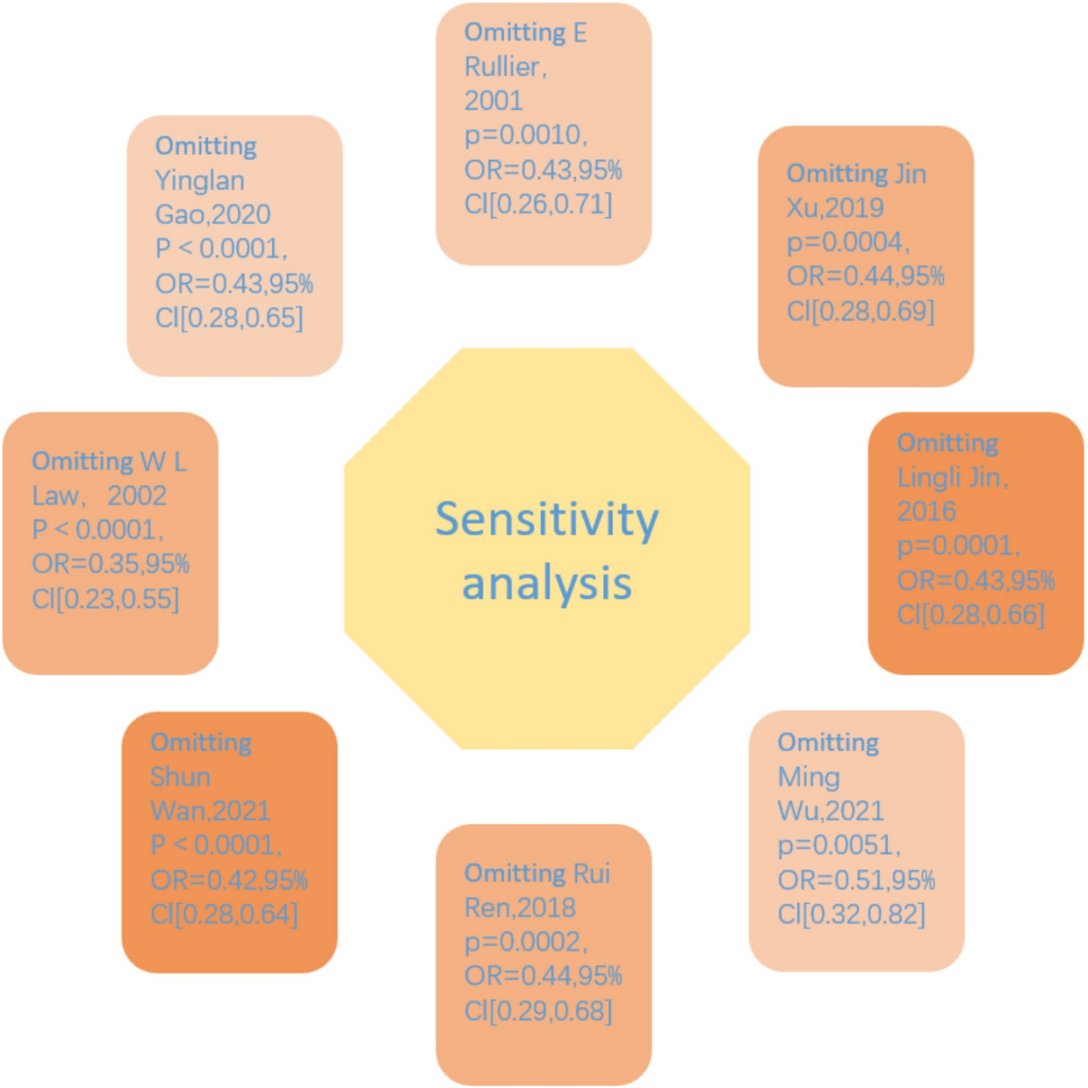
Sensitivity analysis.

To assess the robustness of our meta-analysis findings, we performed a leave-one-out sensitivity analysis by iteratively excluding each study and recalculating the pooled odds ratio (OR). The results demonstrated consistent effect estimates across all iterations, with the pooled ORs ranging from 0.35 (95% CI: 0.23–0.55) to 0.51 (95% CI: 0.32–0.82) (Figure 6). Notably, the exclusion of Law et al. (9)—which showed an opposing effect direction (OR = 6.17) in the primary analysis—yielded the most pronounced change in the pooled estimate (OR = 0.35, 95% CI: 0.23–0.55). Nevertheless, all recalculated ORs remained statistically significant (*p* < 0.01 for all comparisons) and below 1.0, confirming that none of the individual studies substantially altered the overall conclusion. Heterogeneity measures remained low to moderate across all sensitivity tests (I^2^ range: 0–26.7%), with complete elimination of heterogeneity (I^2^ = 0%, τ^2^ = 0) observed upon exclusion of Law et al. (9). The consistency of these results supports the reliability of our primary findings.

## Discussion

### Conclusion

Based on the above data analysis, we can draw the conclusion that in terms of reducing the overall complications of enterostomy surgery, ileostomy has more advantages compared to colostomy. We analyze that this phenomenon may be related to the following aspects:

1. Anatomical and physiological characteristics: The diameter of the ileum is relatively thin and the intestinal wall is relatively thin, making it easier to perform surgical operations and anastomosis during stoma creation, and the incidence rate of complications such as stoma stenosis after surgery is relatively low. The peristaltic function of the ileum is relatively strong, and the intestinal contents stay in the ileum for a relatively short time, which is not easy to accumulate locally, reducing the risk of complications such as infection caused by the retention of intestinal contents (10).
2. The contents of the ileum are usually liquid and have a relatively thin texture, which causes relatively little irritation to the skin around the stoma, reducing the incidence of complications such as peristomal dermatitis. The contents of the colon contain more fecal residues and bacteria, and its excreta are relatively thick, which is likely to adhere around the stoma, increasing the risk of local infection and skin damage (11).
3. Selection of stoma location: Ileostomy is usually located in the right lower abdomen, where the skin is relatively flat, making it convenient for the pasting and fixation of the ostomy bag, and reducing the complications caused by the insecure wearing of the ostomy bag or local friction. The location of the colostomy is relatively diverse, which may be in the left lower abdomen or other parts. The skin in some locations has more folds, increasing the difficulty of fitting the ostomy bag, and prone to problems such as leakage, which may further lead to complications (12).
4. Related to surgical operations: Ileostomy surgery begins with preoperative stoma site marking, typically in the right lower abdomen over the rectus abdominis, avoiding skin folds, scars, or bony prominences to facilitate post-operative ostomy bag attachment. A small incision (about 3–5 cm) is made at the marked site, and the layers of skin, subcutaneous tissue, and the anterior sheath of the rectus abdominis are dissected sequentially to expose the peritoneum. A segment of the terminal ileum (approximately 10–15 cm) is then mobilized, ensuring adequate blood supply and no excessive tension. The ileal end is pulled through the abdominal incision by about 2–3 cm, and its seromuscular layer is sutured to the peritoneum, anterior rectus sheath, and skin layer by layer to secure it. Finally, the distal end of the ileum is everted to form a small “nipple” shape, ensuring unobstructed drainage of intestinal contents. This procedure can also be performed minimally invasively using laparoscopy or robotic assistance, where key steps like intestinal mobilization and stoma formation are completed under endoscopic visualization, with only a small incision needed to bring the ileum out. For colostomy, the stoma is mostly placed in the rectus abdominis in the left lower abdomen or left iliac fossa. For some patients, the position needs to be adjusted due to anatomical variations of the colon, which increases the complexity of preoperative evaluation. After entering the abdominal cavity through an incision in the left lower abdomen, the sigmoid colon and its mesentery need to be mobilized. Since the vascular branches of the colonic mesentery are relatively complex, careful ligation of blood vessels is required to avoid bleeding, and the mobilization process takes longer. After the proximal colon is pulled out of the abdominal wall, the risk of adhesion between the intestinal tube and surrounding tissues needs to be addressed. In addition, the colonic wall is relatively thick and its blood supply is relatively scattered. It is necessary to ensure uniform suture tension during anastomosis; otherwise, leakage may easily occur. Similarly, colostomy can also be performed via laparoscopic or robot-assisted surgery. For procedures involving complex locations such as transverse colostomy, the visual advantage of laparoscopy helps reduce the risk of injury to adjacent organs. Robot-assisted surgery is suitable for patients requiring delicate mesenteric management or reoperation (e.g., abdominal adhesions). The tremor-filtering technology of the robotic system can minimize operational errors and improve suture precision, though the surgical duration may be slightly longer. In summary, Whether it is ileostomy or colostomy, the application of minimally invasive techniques (laparoscopic or robot-assisted) should be determined based on the patient’s condition (such as tumor stage and the degree of intra-abdominal adhesions), the surgeon’s experience, and the availability of medical equipment. Ileostomy surgery is relatively simple, usually with a shorter operation time, less trauma to the patient, and relatively faster postoperative recovery, which can reduce the risk of various complications caused by surgical trauma (8, 9).Colostomy surgery involves a more complex process of intestinal mobilization and anastomosis, with relatively greater surgical difficulty, a longer operation time, and a relatively higher risk of complications such as anastomotic leakage (Caused by insufficient blood supply) and bleeding (Caused by insecure vascular ligation) after surgery.

Moreover, we compared the operation time and postoperative hospital stay mentioned in Table 1. We can find that the operation time for ileostomy is generally shorter than that for colostomy. This indicates that in terms of the difficulty of the operation, ileostomy is simpler than colostomy. Similarly, we can also consider that the ileostomy surgery has a relatively high level of safety. In terms of the postoperative hospital stay, ileostomy also has obvious advantages. Although we are not clear about how much each patient has actually spent, we can indirectly assume that patients with a shorter hospital stay have lower expenses.

In terms of quality of life, both patients with ileostomy and those with colostomy may develop various psychological problems due to the changes in their body structures. However, there are also some differences in quality of life between patients who undergo these two procedures. For example, the effluent from an ileostomy is usually liquid or semi-liquid, with a relatively large volume and fast flow rate, which can easily irritate the skin around the stoma and increase the risk of dermatitis or erosion. Consuming high-fiber, gas-producing, or irritating foods (such as beans, onions, and spicy foods) may lead to increased stool output, diarrhea, or abdominal distension, thereby affecting the stability of the stoma. Nevertheless, our research shows that ileostomy has more advantages in reducing overall stoma complications. We believe that changing the ostomy bag once more a day is not unacceptable compared to increasing the incidence of severe complications such as stoma ischemic necrosis, stoma stenosis, and stoma prolapse. In general, we believe that doctors need to fully communicate with patients about the complications of the two procedures, and seek a balance between treatment effects and quality of life based on medical factors such as the patient’s condition, personal wishes, and surgical indications. Therefore, completely solving the problem of intestinal diversion is the key for patients to regain their confidence. For this reason, Francesk Mulit and others explored the advantages and disadvantages of perineal colostomy. They believed that perineal colostomy not only effectively reduces the probability of complications, but also, because the discharge channel of intestinal contents is close to the natural passage, it effectively ensures the dignity and quality of life of patients. The disadvantage of this method is that the stoma is difficult to manage (13). Therefore, although perineal colostomy has many benefits, doctors also need to be aware of its limitations and reach an agreement with the patients. Due to all the above reasons, the concept of an artificial anus has also emerged (14). However, the existing artificial anuses on the market have various drawbacks. For example, excessive pressure on the clamped intestinal tract may lead to intestinal necrosis, and a too-long opening time makes them inconvenient to use (15). As a result, patients have an increasing demand for a new type of artificial anus. Our team is also conducting relevant research in this regard and has achieved initial results, obtaining encouraging outcomes.

In conclusion, we believe that for patients with intestinal stomas, a new type of effective in-situ artificial anus will bring great benefits to them. However, before that, surgeons can perform ileostomy surgery, which has fewer complications, to relieve the patients’ pain.

### Prevention of complications

Although choosing an appropriate surgical procedure can avoid some complications in terms of preventing and reducing the probability of complications, we can also reduce the incidence of enterostomy complications through other means.

Before the operation, first of all, we need to conduct a comprehensive assessment of the patient’s overall health status, including the functions of important organs such as the heart, lungs, liver, and kidneys, as well as the nutritional status and coagulation function. For patients with malnutrition conditions such as anemia and hypoalbuminemia, nutritional support treatment should be given first to correct anemia and hypoalbuminemia, so as to improve the patient’s tolerance to the operation and the ability of postoperative wound healing. Secondly, we should do a good job in intestinal cleansing preparation. Oral laxatives such as compound polyethylene glycol electrolyte powder can be used to reduce the number of bacteria in the intestine and lower the risk of postoperative infection. At the same time, appropriate antibiotics should be given before the operation for intestinal antibacterial treatment to further prevent infection. Finally, when positioning the stoma, we need to jointly determine the stoma location by experienced doctors and stoma therapists according to the patient’s condition, body shape, activity habits and other factors (12, 16). Generally, it is selected at the rectus abdominis, and areas such as scars, skin folds, and bone protrusions should be avoided to facilitate stoma care and reduce the occurrence of complications.

During the operation, when mobilizing the intestinal tract, attention should be paid to protecting the blood supply of the intestinal tract and avoiding damage to the mesenteric vessels to ensure good blood supply to the stoma intestinal segment and prevent complications such as intestinal ischemia and necrosis. When bringing the intestinal tract out of the abdominal wall, attention should be paid to maintaining an appropriate tension. It should not be too tight to cause compression and ischemia of the intestinal tract, nor too loose to form intestinal loop sagging, which will affect intestinal peristalsis and drainage of excreta. The fixation of the stoma intestinal tract and the abdominal wall should be firm to prevent the intestinal tract from retracting or prolapsing. Generally, a multi-layer suture fixation method is used to ensure a tight fit between the intestinal tract and the abdominal wall and reduce the chances of leakage and infection.

During the postoperative nursing process, we need to keep the skin around the stoma clean and dry, change the ostomy bag in a timely manner, and avoid the excreta from irritating the skin and causing redness, swelling, ulceration, etc. Nursing supplies such as stoma skin care powder and skin protective film can be used to protect the skin around the stoma. Pay attention to observing the color and shape of the stoma and the properties and quantity of the excreta. In terms of diet, stoma patients should fast in the early postoperative period and gradually start eating after the intestinal function recovers. Start with liquid and semi-liquid foods and gradually transition to a normal diet. Avoid eating spicy, greasy, and irritating foods, as well as foods that are likely to produce gas and are difficult to digest, such as beans, onions, glutinous rice, etc., to prevent discomfort such as abdominal distension and diarrhea (17).

In addition, in terms of postoperative nursing, the most important thing is the health education for patients, which includes the following points: 1. Encourage patients to turn over and move their limbs in bed in the early stage to promote intestinal peristalsis and blood circulation. After the physical condition permits, gradually increase the amount of activity, but avoid strenuous exercise and actions that increase abdominal pressure, such as lifting heavy objects and straining during defecation, to prevent stoma prolapse or parastomal hernia (18). 2. Enterostomy may bring psychological pressure and inconvenience to patients. In severe cases, patients may even have the idea of committing suicide due to discrimination from people around them. Medical staff should communicate more with patients, understand their psychological state, and provide psychological support and comfort. At the same time, stoma rehabilitation patients can be invited to share their experiences to help patients build confidence and face life positively (19). 3. Instruct patients to regularly visit the hospital for reexaminations, including stoma examinations, abdominal ultrasound, blood routine, etc., so as to detect and deal with possible complications in a timely manner, such as stoma stenosis, intestinal obstruction, infection, etc. Doctors can also give corresponding rehabilitation guidance and suggestions according to the patient’s recovery situation (20).

According to different types of enterostomy complications, we can also adopt different measures to reduce the incidence of complications. In terms of reducing enterostomy bleeding, the surgeon should carefully stop the bleeding during the operation to ensure that the blood vessels around the stoma are firmly ligated. For ileostomy, attention should be paid to the treatment of the mesenteric vessels; when selecting the location of the colostomy, the area with abundant blood vessels should be avoided. In terms of reducing the risk of infection, strictly follow the principle of aseptic operation, protect the tissue around the stoma, and avoid the contamination of the abdominal cavity and the incision by intestinal contents. For colostomy, attention should be paid to closing the mesenteric hiatus during the operation to prevent the formation of internal hernia leading to infection. In terms of reducing the incidence of parastomal hernia, a patch can be used prophylactically (21).

**Figure 7:**
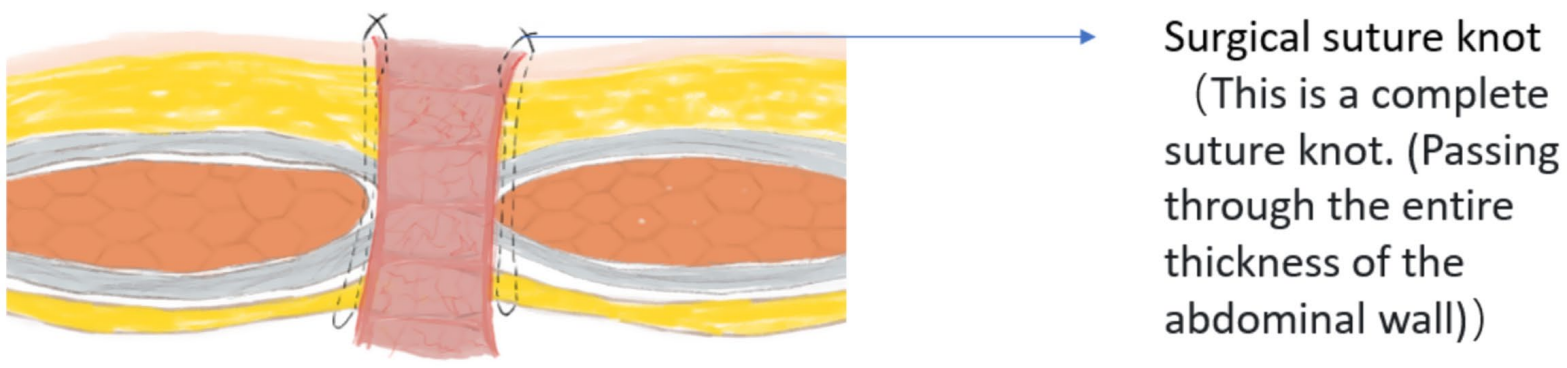
The methods used by doctors in a certain country to fix intestinal stomas.

**Figure 8:**
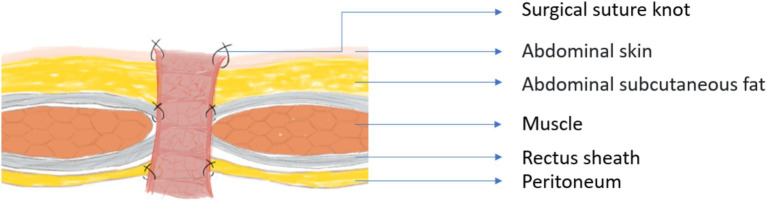
Methods used by Chinese doctors to fix intestinal stomas.

In addition, during the process of retrieving articles, we found that in most studies from China, the probability of parastomal hernia is very low. However, in studies not from China, we found that the incidence of parastomal hernia even ranks first or second among enterostomy complications. We speculate that this may be related to the surgical operation method. During the period when the corresponding author of this article served as a visiting scholar in a certain country, it was observed that doctors usually choose to directly penetrate the abdominal wall and the seromuscular layer of the intestinal tract with a single needle when fixing the enterostomy, as shown in Figure 7. Then, the surgeon will use the same method as described above to sew the remaining 7 stitches around the intestinal stoma. In fact, due to the limitation of the suture needle size, it remains uncertain whether this suturing method can achieve the goal of penetrating the entire thickness of the abdominal wall. Therefore, we believe this might be one of the reasons for the high incidence of parastomal hernia. Moreover, even if it is possible to penetrate the entire thickness of the abdominal wall when fixing the stoma, the limited surgical field will increase the risk of the suture needle puncturing the intestinal tract. However, in China, doctors usually choose to fix the intestinal wall to the peritoneum, rectus sheath, and skin in sequence from bottom to top, as shown in Figure 8. In this process, at least 8 stitches are made for each layer of structure, and even 20 or more stitches are made when fixing the skin and the intestinal wall together. We believe that these two different surgical operation styles may lead to different incidences of parastomal hernia. However, whether this surgical method of Chinese doctors will lead to the occurrence of enterostomy ischemia remains to be investigated.

### Limitation

In this study, the random-effects model was used to combine the data. Although it can provide a reference for clinicians to select surgical procedures, there are significant differences among the studies in terms of stoma type (colon/ileum), surgical method (open/laparoscopic), follow-up time, and complication assessment criteria. Residual confounding factors may affect the reliability of the results. Secondly, the follow-up time of the study is less than 5 years, making it difficult to assess the true incidence rate of delayed complications (such as stoma-related squamous cell carcinoma), and original studies with a longer follow-up period are needed for supplementation. Finally, the number of included studies and patients is relatively small, which may have a slight impact on the results.

## Data Availability

The datasets presented in this study can be found in online repositories. The names of the repository/repositories and accession number(s) can be found in the article/supplementary material.
